# A Stress-Responsive Signaling Network Regulating Pseudohyphal Growth and Ribonucleoprotein Granule Abundance in *Saccharomyces cerevisiae*

**DOI:** 10.1534/genetics.119.302538

**Published:** 2019-08-27

**Authors:** Nebibe Mutlu, Daniel T. Sheidy, Angela Hsu, Han Seol Jeong, Katherine J. Wozniak, Anuj Kumar

**Affiliations:** * Department of Molecular, Cellular, and Developmental Biology, University of Michigan, Ann Arbor, Michigan 48109; † Program in Molecular and Cellular Biology, University of Michigan, Ann Arbor, Michigan 48109

**Keywords:** yeast, functional genomics, proteomics, pseudohyphal growth

## Abstract

The budding yeast *Saccharomyces cerevisiae* undergoes a stress-responsive transition to a pseudohyphal growth form in which cells elongate and remain connected in multicellular filaments. Pseudohyphal growth is regulated through conserved signaling networks that control cell growth and the response to glucose or nitrogen limitation in metazoans. These networks are incompletely understood, and our studies identify the TORC1- and PKA-regulated kinase Ksp1p as a key stress-responsive signaling effector in the yeast pseudohyphal growth response. The kinase-defective *ksp1*-K47D allele results in decreased pseudohyphal morphology at the cellular and colony level, indicating that Ksp1p kinase signaling is required for pseudohyphal filamentation. To determine the functional consequences of Ksp1p signaling, we implemented transcriptional profiling and quantitative phosphoproteomic analysis of *ksp1*-K47D on a global scale. Ksp1p kinase signaling maintains wild-type transcript levels of many pathways for amino acid synthesis and metabolism, relevant for the regulation of translation under conditions of nutrient stress. Proteins in stress-responsive ribonucleoprotein granules are regulated post-translationally by Ksp1p, and the Ksp1p-dependent phosphorylation sites S176 in eIF4G/Tif4631p and S436 in Pbp1p are required for wild-type levels of pseudohyphal growth and Protein Kinase A pathway activity. Pbp1p and Tif4631p localize in stress granules, and the *ksp1* null mutant shows elevated abundance of Pbp1p puncta relative to wild-type. Collectively, the Ksp1p kinase signaling network integrates polarized pseudohyphal morphogenesis and translational regulation through the stress-responsive transcriptional control of pathways for amino acid metabolism and post-translational modification of translation factors affecting stress granule abundance.

THE eukaryotic cellular response to nutrient limitation is complex, encompassing programmed changes resulting in reduced protein translation, increased autophagy, an elevated abundance of ribonucleoprotein (RNP) granules, altered anabolism and catabolism of metabolites, and, in some organisms, pronounced morphological changes (Gimeno *et al.* 1992; Gasch *et al.* 2000; Klionsky and Emr 2000; Parker and Sheth 2007). In certain strains of the budding yeast *Saccharomyces cerevisiae*, nitrogen or glucose limitation induces a reversible transition from a yeast-like unicellular form to a filamentous or pseudohyphal morphotype, characterized by the formation of multicellular filaments (Gimeno *et al.* 1992; Cullen and Sprague 2000). Cells undergoing pseudohyphal growth bud distally in a unipolar fashion, exhibit a polarized actin cytoskeleton, and remain physically connected after cytokinesis (Gimeno and Fink 1994; Kron *et al.* 1994; Lo and Dranginis 1996; Li and Mitchell 1997; Reynolds and Fink 2001; Cullen and Sprague 2002). Filamentation in *S. cerevisiae* manifests itself principally in two growth forms affected by strain ploidy and nutrient availability. In haploid invasive growth, yeast colonies grown on standard or rich medium form filaments that extend downward, invading agar and other semisoft surfaces (Li and Mitchell 1997; Mösch *et al.* 1999; Cullen and Sprague 2000, 2002). In diploid pseudohyphal growth, nitrogen or glucose limitation induces the formation of filaments that extend downward from the colony into the substrate and outward over the surface of the agar (Gimeno *et al.* 1992; Cullen and Sprague 2012; Shively *et al.* 2013). Some induction stimuli, including the presence of short-chain alcohols that mimic amino acid catabolism observed during nitrogen limitation, can induce both surface filamentation and invasion in haploid yeast (Lorenz *et al.* 2000). Filament formation is thought to constitute a scavenging mechanism, and a pseudohyphal strain of *S. cerevisiae* grows in culture to a higher optical density than a wild-type, nonfilamentous strain under conditions of extended nitrogen limitation (Gimeno *et al.* 1992; Ma *et al.* 2007). Filamentation in *S. cerevisiae* is an informative model of pseudohyphal and hyphal development in the related opportunistic human fungal pathogen *Candida albicans* (Braun and Johnson 1997; Saville *et al.* 2003; Nobile and Mitchell 2005; Whiteway and Bachewich 2007). Filamentous development in *C. albicans* is required for pathogenicity in a mouse model of disseminated candidiasis (Lo *et al.* 1997). Yeast pseudohyphal growth has also been intensely studied as a tractable model of highly conserved signaling pathways that regulate metazoan cell growth and stress responses (Minden *et al.* 1994; Beck and Hall 1999; Westfall *et al.* 2008; Sengupta *et al.* 2010; Basu *et al.* 2016).

Landmark work from numerous groups have identified signaling pathways required for wild-type pseudohyphal growth, including the Kss1p mitogen-activated protein kinase (MAPK) cascade (Liu *et al.* 1993; Zhou *et al.* 1993; Roberts and Fink 1994; Cook *et al.* 1996; Madhani and Fink 1997; Erdman *et al.* 1998; Erdman and Snyder 2001), the rat sarcoma/cAMP-dependent protein kinase A pathway (Pan and Heitman 1999, 2002; Stanhill *et al.* 1999), the target of rapamycin (TOR) network (Cutler *et al.* 2001; Loewith *et al.* 2002; Braus *et al.* 2003), and the AMP-activated/sucrose nonfermentable (AMPK/Snf1p) pathway (Kuchin *et al.* 2002, 2003). Points of crosstalk between these networks can identify important signaling nodes (Borneman *et al.* 2006), and in this light, the yeast kinase Ksp1p is notable, as recent studies suggest that it is regulated through the PKA and TOR pathways. Ksp1p, a protein of the casein kinase II subfamily, was identified by mass spectrometry in a complex purified by immunoprecipitation of the core TOR Complex 1 protein Kog1p (Laxman and Tu 2011). Ksp1p contains PKA consensus recognition motifs (residues Ser591, Ser624, Ser827, and Ser884) and exhibits decreased phosphorylation at Ser827 and Ser884 upon rapamycin treatment (Soulard *et al.* 2010). Ksp1p was first identified as a high-copy suppressor of a temperature-sensitive mutation (prp20*-10*) in the *SRM1*/*PRP20* gene encoding a nucleotide exchange factor for Ran/GSP1 proteins (Fleischmann *et al.* 1996). We identified *KSP1* in a loss-of-function screen for genes required for wild-type filamentous growth in a haploid derivative of the Σ1278b strain under conditions of butanol induction (Jin *et al.* 2008). We found that *KSP1* was required for the wild-type localization of several pseudohyphal growth signaling proteins, including the Bcy1p regulatory subunit of PKA (Bharucha *et al.* 2008). Subsequently, Umekawa and Klionsky (2012) reported that *KSP1* negatively regulates autophagy, consistent with the observed hypo-phosphorylation of Atg13p in *ksp1*Δ, and that this suppressive function of Ksp1p is partially activated by PKA.

These studies suggest that *KSP1* contributes to eukaryotic cell signaling through stress-responsive pathways that regulate pseudohyphal growth, among other cell processes; however, the functional significance of Ksp1p kinase signaling is unclear, and the scope of the Ksp1p kinase signaling network remains to be determined. Here, we present data indicating the relevance of Ksp1p kinase activity in the yeast pseudohyphal response and globally identify changes in transcript abundance and protein phosphorylation dependent upon Ksp1p kinase signaling under filamentation-inducing conditions. The data identify phosphorylation sites in translation-associated RNP granule proteins that yield pseudohyphal growth phenotypes upon site-specific mutation. We assess the function of Ksp1p signaling in regulating RNP granule abundance and indicate that loss of *KSP1* results in elevated Pbp1p-marked granules. Collectively, the work identifies Ksp1p as part of the mechanism linking highly conserved nutrient stress-responsive signaling pathways with the regulation of RNP granules, while identifying the molecular basis of Ksp1p kinase signaling as a TORC1 and PKA downstream effector.

## Materials and Methods

### Strains, plasmids, and media

Filamentous yeast strains were derived from the genetic background Σ1278b. Haploid strains were derived from HLY337 and Y825 (Xu *et al.* 2010). Yeast cells were cultured according to standard techniques as described previously (Guthrie and Fink 1991; Song *et al.* 2014). Yeast cells were grown in YPD (2% peptone, 1% yeast extract, 2% glucose, and 2% agar as needed) or synthetic complete (SC) medium (0.67% yeast nitrogen base, 2% glucose, 0.2% of the appropriate amino acid dropout mix, and 2% agar, as needed). Low-nitrogen synthetic low-ammonia dextrose (SLAD) media was prepared as follows: 0.17% yeast nitrogen base without amino acids and without ammonium sulfate, 2% glucose, 50 μM ammonium sulfate, and appropriate amino acids to complement auxotrophy. Synthetic low-ammonium low-dextrose (SLALD) media was prepared as described for SLAD media but with 0.05% glucose (Johnson *et al.* 2014).

The plasmid pDS7 (U1A-mCherry) was constructed by amplification of the mCherry protein from plasmid pBS35 (Hailey *et al.* 2002) using the oligonucleotide primers DSK116 and DSK117, and amplification of the pRP1194 backbone (Buchan *et al.* 2010) using oligonucleotide primers DSK114 and DSK115. PCR products were used for Gibson assembly with Gibson Assembly Master Mix (New England Biolabs, Beverly, MA).

### Construction of chromosomal point mutants and gene fusions

Chromosomal deletions of genes were generated using a one-step PCR-based protocol for gene disruption by amplifying the *kanMX6* sequence from plasmid pFA6a-*kanMX6* (Longtine *et al.* 1998). Transformants were selected by plating on YPD medium supplemented with G418. PCR was used to confirm integration of the insertion cassette. Carboxy-terminal GFP fusions were generated by transformation of relevant strains with PCR product after amplification of the *GFP-HisMX6* cassette from pFa6a-GFP-HisMX6 (Longtine *et al.* 1998). Chromosomal point mutants were generated using a *URA3*-based gene replacement and counter-selection strategy as described previously (Gray *et al.* 2005; Ma *et al.* 2008). In brief, the *URA3* gene was amplified from pRS406 with primers containing sequence corresponding to 45-bp regions on either side of the mutational target site. Strains were transformed with PCR product for selection on media lacking uracil. Strains with *URA3* insertions were transformed with annealed 120-nt oligonucleotides containing the desired mutant sequence and homology to flanking regions. Loss of *URA3* was monitored by counterselection on plates supplemented with 5-fluoroorotic acid.

### Surface-spread pseudohyphal growth and invasion assays

Cultures of diploid wild-type or mutant strains were grown overnight in standard growth media (*e.g.*, YPD media) before being diluted 1:20 in fresh media. Strains were subsequently grown for 4–6 hr prior to harvesting for plating. Cells were washed three times with sterile deionized water and normalized to a final optical density of 1.0 before being diluted and spread onto plates with SLAD medium supplemented with uracil or other amino acids to complement strain auxotrophy. Cells were spread at a density of ∼50 cells/plate. Plates were incubated at 30° until filamentation was observed in wild-type strains, and colonies were then imaged using an upright Nikon Eclipse 80i microscope with CoolSnap ES2 CCD (Photometrics). Surface filamentation was quantified as described (Ryan *et al.* 2012; Norman *et al.* 2018). For these analyses, the circumference of a defined area of a colony was measured using ImageJ and compared against the circumference of the same defined area of a wild-type colony. The ratio is calculated from three replicates, and the average and SD are indicated.

Agar invasion was calculated by standard protocols (Ryan *et al.* 2012; Shively *et al.* 2013; Norman *et al.* 2018). A 5 μl aliquot of the culture to be tested was spotted onto a plate and allowed to invade the agar for 2–3 days. Plates were photographed, and surface cells were washed under a gentle stream of water. The washed cultures were imaged again, and the degree of invasive growth was quantified as the mean pixel intensity of the washed spot relative to its previous image. Triplicate replicates were assayed for each strain. To analyze cell morphology, cells were scraped from the edge of a colony and resuspended in a small volume of media before spotting onto a slide for microscopy. Images were captured as described above. Cell dimensions were measured, and height-to-width ratios for individual cells were calculated. Cells with a height-to-width ratio ≥2 were indicative of pseudohyphal growth.

### Transcriptional profiling and analysis

Single colonies of respective strains (yCK186 and DSY005) were inoculated in YPD media and grown overnight with shaking at 30°. Subsequently, cells corresponding to an optical density (600 nm) of 1.25 were harvested at 4000 g for 4 min and suspended in 5 ml SLAD media supplemented with uracil, yielding a final OD_600_ of 0.25/ml. Cultures were grown for an additional 6 hr in SLAD media supplemented with uracil. Cells were then harvested and RNA isolated using the RiboPure Yeast Kit (Invitrogen, Carlsbad, CA) according to manufacturer instructions. RNA concentration was determined using the NanoDrop microvolume spectrophotometer, and the quality of prepared RNA was assessed at the University of Michigan Sequencing Core with the BioAnalyzer platform (Agilent). Libraries for sequencing were prepared according to standard protocols (Song *et al.* 2014), and sequencing was performed with the Illumina HiSeq 4000 Single-End 51 Cycle platform. Differential transcript abundance at the gene and isoform level was determined using DESeq2 and the Tuxedo pipeline (Trapnell *et al.* 2013; Love *et al.* 2014). Lists of differentially abundant transcripts identified by DESeq2 and Tuxedo are available as Supplemental Material. Diagnostic plots for the sequencing generated by each analysis method can also be accessed as Supplemental Material.

Transcripts significantly increased or decreased according to analysis by both DESeq2 and Tuxedo were investigated further for overrepresented biological processes using DAVID 6.8 (Huang da *et al.* 2009). Enriched gene ontology (GO) terms were summarized using the REVIGO software (Supek *et al.* 2011).

### Mass spectrometry and analysis

Wild-type control and *ksp1*-K47D mutant cells were isotopically labeled with heavy (Lys-8/Arg-10) amino acids in culture. Cell cultures were lysed by bead beating as described previously (Johnson *et al.* 2014; Shively *et al.* 2015). Extracted proteins were quantified using the Bradford assay. Equal quantities of proteins from triplicate independent cultures for each strain were treated for disulfide reduction and alkylation; treated protein preparations were digested with N-tosylamidophenylethyl methyl ketone (TPMK)-treated trypsin (Worthington Biochemical, Lakewood, NJ). Peptide mixtures were fractionated by strong cation exchange on a PolySulfoethyl A column (150 × 4 mm; PolyLC). Fractionation protocols are as described (Johnson *et al.* 2014). Collected fractions were enriched for phosphopeptides using ZrO2 (50 μm inner diameter resin; Glygen). Eluates of enriched phosphopeptides and flow-through from each cation fractionation were analyzed by nano-liquid chromatography–tandem mass spectrometry on a hybrid type mass spectrometer (LTQ-Orbitrap XL; Thermo Fisher Scientific, Waltham, MA) coupled to a nanoLC system (2D nanoLC; Eksigent).

Raw mass spectrometry data were processed and quantified using MaxQuant and the Mascot search engine collectively (Eng *et al.* 1994; Cox *et al.* 2009). Mascot searches were performed against a composite database of forward and reverse sequences of verified yeast open reading frames from the *Saccharomyces* Genome Database. Variable modifications were allowed for oxidation (M) and phosphorylations (STY), as well as a fixed modification of carbamidomethylation (C). Peptide, protein, and phosphorylation site identifications were filtered at a false discovery rate of 5%. The MaxQuant normalized heavy:light ratios with significance scores <0.05 were considered significant in this study. The data were further filtered to exclude peptides exhibiting low Mascot scores (<3), high charge states (>5), and long peptide lengths (>40). Proteins were analyzed for enrichment of associated biological functions using tools made available through the *Saccharomyces* Genome Database, as well as the DAVID software suite (Huang da *et al.* 2009). Protein-protein interaction networks were generated using Cytoscape software (Killcoyne *et al.* 2009), and interactions used to construct the network diagram were extracted from BioGRID.

### Reporter assays of FLO11 transcription

Diploid strains harboring plasmid pGL669-Z FLO11 6/7 (Aun *et al.* 2013) were grown overnight in SC media lacking uracil. Cells were washed three times with 2% glucose, diluted 1:5 in SLAD media, and grown at 30° for 4–6 hr. β-galactosidase assays were performed in triplicate using the Yeast β-Galactosidase Assay Kit (Thermo Scientific) according to manufacturer suggested protocols.

### Fluorescence microscopy

Liquid cultures from single colonies were grown in appropriate media overnight. Cultures were subsequently diluted to an optical density (600 nm) of 0.1 and were grown for 24 or 48 hr. Aliquots (1 ml) of the cultures were removed and cells collected by centrifugation at 1000 × *g* for 1 min. Cells were suspended in 80–100 μl of appropriate SC-based media for imaging using the Deltavision Spectris system (Applied Precision).

### Statistical analysis and error correction

The significance of transcriptional profiling data analyzed by DESeq2 was determined using the Wald test *P*-value for condition *vs.* control analysis. This test statistic *P*-value was adjusted using the Benjamini–Hochberg correction as indicated in File S2. Data analysis through the Tuxedo pipeline determines the uncorrected *P*-value of the test statistic and its false discovery rate–adjusted *P*-value. Both sets of values are reported in File S4. Quantitative phosphoproteomic data were filtered at a false discovery rate of 5%, and MaxQuant-normalized heavy:light ratios for observed peptides were considered statistically significant at a significance score <0.05 (File S7). GO term enrichment in the set of differentially phosphorylated proteins identified by mass spectrometry was analyzed using the DAVID software suite; adjusted *P*-values for enriched GO terms were calculated with the Bonferroni correction and the Benjamini–Hochberg procedure, as indicated. For cell morphology and microscopy data, mean values of the control and test samples were measured, and uncorrected *P*-values were generated using *t*-tests for the comparison of observed means in two independent samples.

### Data availability

Strains and plasmids are available upon request. Supplemental Material, Table S1 lists genotype and source for all strains used in this study. Figure S1 presents phenotypes for *ksp1* deletion and kinase-defective mutants under pseudohyphal growth-inducing conditions of nitrogen limitation and limited glucose availability (SLALD medium). Figure S2 presents images indicating that Pbp1p-GFP and U1A-mCherry-bound RNA puncta are elevated in numbers relative to wild type in a homozygous diploid strain deleted for *KSP1*. Figure S3 presents a diagram of the P_FLO11-6/7_*-lacZ* reporter and indicates reporter activity in a yeast strain deleted for *FLO8*. Figure S4 presents Western blots indicating that protein levels of ste20p-T203A, tif4631p-S176A, and pbp1-S436A are not significantly different from levels of corresponding wild-type proteins; this further suggests that observed phenotypes for these point mutants do not result from altered expression relative to wild type. File S1 contains detailed descriptions of all supplemental files. File S2 contains statistical analysis of all changes in transcript abundance, both significant and insignificant, between the *ksp1*-K47D mutant and wild type as determined using DESeq2. File S3 contains boxplots indicating non-normalized counts, depth-normalized counts, and regularized log2-normalized counts for each RNA-sequencing sample analyzed. File S4 contains statistical analysis of all changes in transcript abundance, both significant and insignificant, between the *ksp1*-K47D mutant and wild type as determined using Tuxedo. File S5 presents summary boxplots of fragments per kilobase of transcript per million mapped reads distribution in log10 scale for each RNA sample. File S6 provides a listing of the union set of genes identified as undergoing statistically significant changes in transcript abundance between *ksp1*-K47D and wild type by both DESeq2 and Tuxedo analysis. File S7 provides the mass spectrometry data from Mascot and MaxQuant analysis for differentially abundant phosphopeptides between *ksp1*-K47D and wild type. File S8 provides the protein-protein interactions extracted from BioGRID for the construction of the network presented in Figure 4. Supplemental material available at FigShare: https://doi.org/10.25386/genetics.8956511.

## Results

### The kinase activity of Ksp1p is required for pseudohyphal growth

*KSP1* encodes a protein of 1029 amino acids, with an estimated molecular mass of 117 kDa. Ksp1p contains a kinase domain, putatively of the casein kinase II family, as part of a strongly conserved region in the amino-terminal half of its protein sequence. The carboxy-terminal half of the protein is enriched in Asn residues and is predicted to bind RNA (Romero *et al.* 2001; He *et al.* 2009). Experimental evidence supports this hypothesis; Mitchell *et al.* (2013) identified Ksp1p as a protein cross-linked to messenger RNA (mRNA). Given the range of functionalities associated with Ksp1p, we sought to determine the contributions of Ksp1p kinase activity toward pseudohyphal growth induced by nutrient limitation.

**Figure 1:**
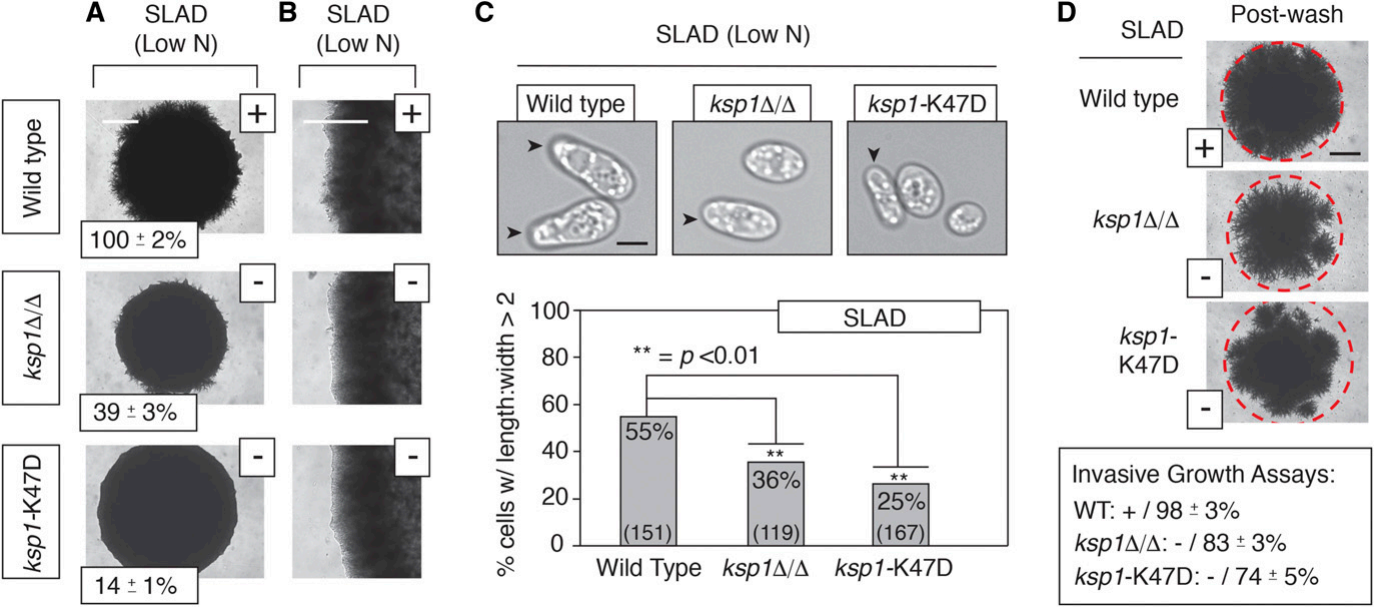
Ksp1p kinase signaling is required for wild-type pseudohyphal growth. (A) Surface filamentation of the indicated strains is shown on medium with low levels of ammonium sulfate (SLAD) as the primary nitrogen source. Filamentation was quantified as the percentage of the circumference for a defined region of the mutant colony relative to the circumference for the corresponding region of a wild-type colony. Percentages indicate the mean of three replicates with SD. All strains are homozygous diploid. + indicates wild-type surface filamentation; − indicates decreased filamentation. Bar, 1 mm. (B) Higher magnification image of a colony edge. Bar, 500 μm. (C) Colony morphology of homozygous diploid wild-type and *ksp1* mutants under pseudohyphal growth-inducing conditions of nitrogen limitation. Arrowheads indicate elongated cells with a length:width ratio >2. The percentage of cells with an elongated morphology is shown for each strain. The number of cells counted is indicated in parentheses at the base of each histogram bar. *P*-values are calculated for pairwise comparisons using two-sample *t*-tests. Bar, 1 μm. (D) Agar invasion of homozygous diploid strains grown on low-nitrogen (SLAD) medium. Invasion was detected by washing away surface cells in a gentle stream of water. The remaining cells were imaged, and the degree of invasion was quantified as the percentage of the average pixel intensity postwashing relative to the average pixel intensity of the unwashed spotted culture. The dashed red circle indicates the circumference of the spotted culture prior to washing. The mean from three replicates with SD is indicated. + indicates wild-type invasion; − indicates decreased invasion relative to wild type. Bar, 2 mm.

To address this, we mutated the conserved catalytic lysine residue (Lys47) in the predicted ATP-binding pocket of the Ksp1p kinase domain for phenotypic analysis of pseudohyphal growth. The Lys-to-Asp substitution was achieved by allelic replacement at the native *KSP1* locus in a strain of the filamentous Σ1278b genetic background, and the homozygous diploid *ksp1*-K47D kinase-defective mutant was assayed for surface-spread filament formation under conditions of nitrogen limitation. As indicated in Figure 1, A and B, the kinase-defective *ksp1*-K47D strain exhibits decreased surface filamentation relative to wild type on medium with low levels of ammonium sulfate as a nitrogen source. This surface filamentation phenotype is also evident in *ksp1*-K47D under pseudohyphal growth conditions of limited nitrogen and reduced glucose availability (SLALD), although the phenotype is less severe than that observed under conditions of limited nitrogen alone (Figure S1). Cells undergoing pseudohyphal growth are elongated, and the percentage of cells with an elongated morphology is significantly decreased in the *ksp1*-K47D strain relative to wild type under conditions of nitrogen limitation (Figure 1C). The cell morphology of *ksp1* mutants in medium with limited nitrogen and glucose is quantified in Figure S1. Agar filament invasion is also diminished in homozygous diploid *ksp1*-K47D on low-nitrogen medium (Figure 1D).

### A global profile of changes in transcript abundance is dependent upon Ksp1p kinase activity

**Figure 2:**
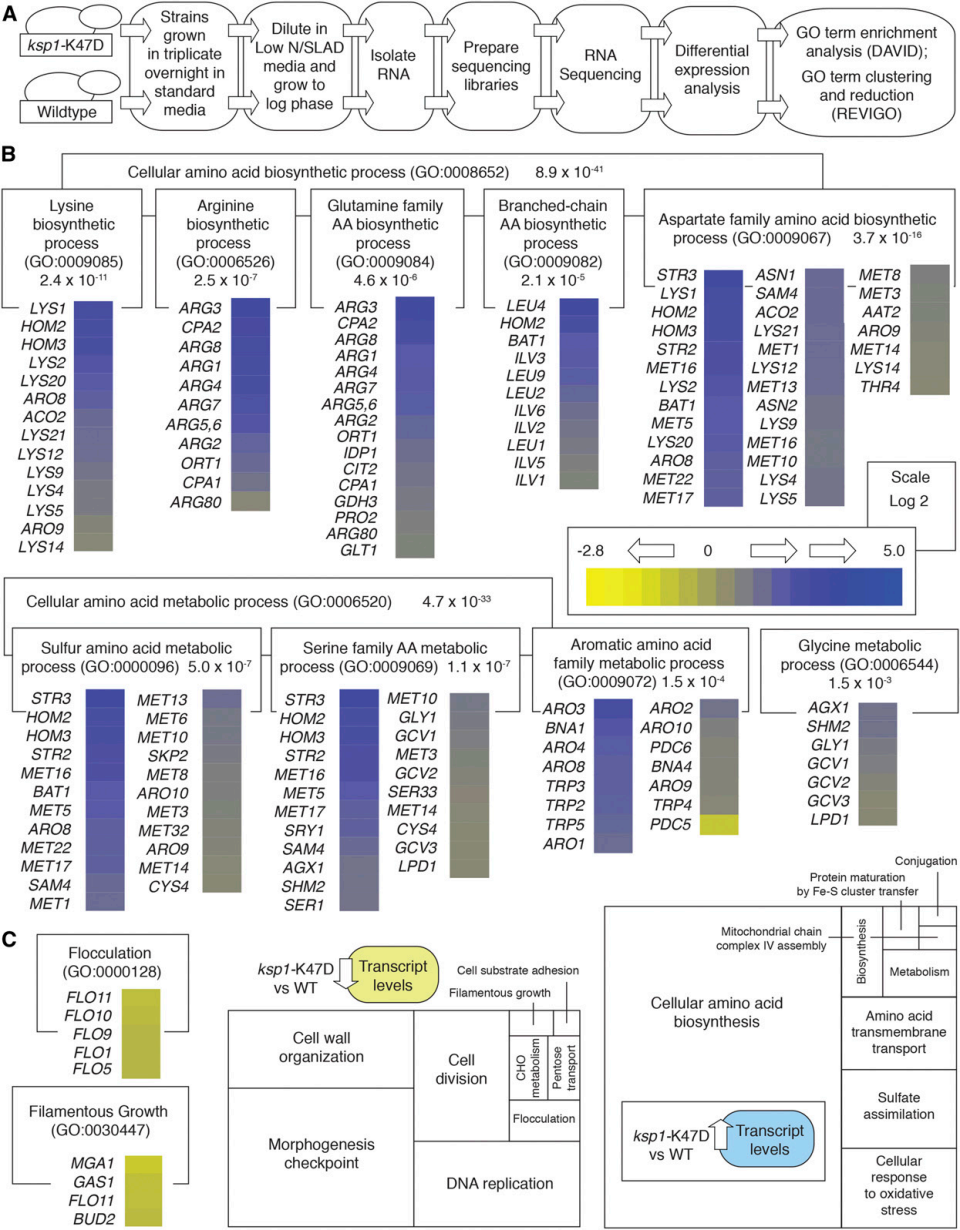
Transcriptional profiling identifies cellular processes affected by Ksp1p kinase signaling. (A) Workflow for the identification and analysis of transcripts differentially abundant in the kinase-defective *ksp1*-K47D strain relative to wild type. (B) Genes exhibiting differentially abundant transcripts were analyzed for enriched GO terms. Cell processes associated with the biosynthesis and metabolism of amino acid pools were enriched in the data set; relevant GO terms with annotated transcripts are indicated. The *P*-value associated with each enrichment is shown. Changes in transcript abundance for each gene are represented by the indicated color scale. Changes in transcript abundance were calculated using the base-2 logarithmic scale. (C) Transcripts for known flocculence (GO: 0000128) and filamentous growth (GO: 0030447) genes were less abundant in *ksp1*-K47D relative to wild type. GO annotations enriched in the set of transcripts exhibiting decreased abundance in *ksp1*-K47D and in the transcripts with increased abundance in this mutant are represented in the boxed diagram. The magnitude of enrichment for each term is indicated by the size of each rectangle, with the full box indicating the sum total of enriched GO terms for the respective data set.

As a step toward determining the molecular basis of Ksp1p kinase signaling in regulating the pseudohyphal response, we profiled changes in transcript abundance in *ksp1*-K47D relative to wild type under conditions of reduced nitrogen availability (SLAD media). Cells were grown to log phase in culture for subsequent harvesting, RNA extraction, and high-throughput sequencing (Figure 2A). Transcripts with altered abundance in *ksp1*-K47D were analyzed for statistically significant enrichment of annotated GO terms and cellular/biochemical properties of the encoded protein products.

In total, we report a union set of 741 transcripts exhibiting altered abundance in the *ksp1*-K47D mutant as identified through analyses using two independent methods (detailed in *Materials and Methods*). Approximately 36% of transcripts exhibited decreased abundance in *ksp1*-K47D relative to wild type, with the remaining 64% of transcripts showing increased abundance. Collectively, transcripts with altered abundance were highly enriched for genes contributing to cellular amino acid biosynthesis and metabolism (Figure 2B); by GO annotation, the latter term encompasses both biosynthetic and catabolic proteins. Affected amino acid biosynthetic and metabolic pathways encompass gene sets responsible for regulating charged/polar amino acids (including lysine, arginine, aspartic acid, glutamine, and sulfur-containing residues such as serine and cysteine), as well as pathways regulating aromatic amino acids and hydrophobic residues (including glycine and branched-chain amino acids). It is also notable that the transcripts annotated as contributing to amino acid biosynthesis or metabolism were predominantly more abundant in the *ksp1*-K47D mutant relative to wild type. Translation initiation and polyribosome abundance are markedly decreased under many stress conditions in eukaryotes, and the results here indicate a role for Ksp1p in decreasing the transcription of genes contributing to cellular amino acid pools as an output of its kinase signaling activity.

Transcripts exhibiting decreased abundance in *ksp1*-K47D were enriched for GO terms associated with the morphogenesis checkpoint, DNA replication, and cell wall organization (Figure 2C). The acute onset of starvation activates morphogenesis and DNA replication checkpoints (Giannattasio and Branzei 2017), and the decrease in transcript abundance for related genes in *ksp1*-K47D may manifest as a defect in the sensing or processing of starvation signals upon impaired Ksp1p kinase signaling. Perhaps expectedly, a significant set of flocculence (*FLO*) genes show decreased transcript abundance in the *ksp1*-K47D mutant under conditions that induce pseudohyphal growth. *FLO* genes are so named because they are required for flocculence, or cell-cell adhesion, which is increased in filamentation-competent strains during pseudohyphal growth (Lo and Dranginis 1998; Halme *et al.* 2004; Karunanithi *et al.* 2010). *FLO11* is required for wild-type pseudohyphal growth, and its complex and unusually large 3-kb promoter is regulated through transcription factors acted upon by the Kss1p MAPK, PKA, and Snf1p signaling pathways (Madhani *et al.* 1999; Rupp *et al.* 1999; Bao *et al.* 2004; van Dyk *et al.* 2005). *FLO11* transcript abundance is diminished in the *ksp1*-K47D strain, consistent with the observed decrease in surface-spread and invasive filamentation evident in this mutant (Figure 2C).

In total, these data are consistent with a function for Ksp1p kinase activity in regulating translation and cell morphogenesis at the transcriptional level. To consider post-translational modifications in phosphorylation achieved directly or indirectly through Ksp1p kinase signaling, we employed an approach using quantitative phosphoproteomics.

### Quantitative phosphoproteomics identifies a set of messenger RNP granule proteins differentially phosphorylated in the ksp1-K47D kinase-defective mutant

**Figure 3:**
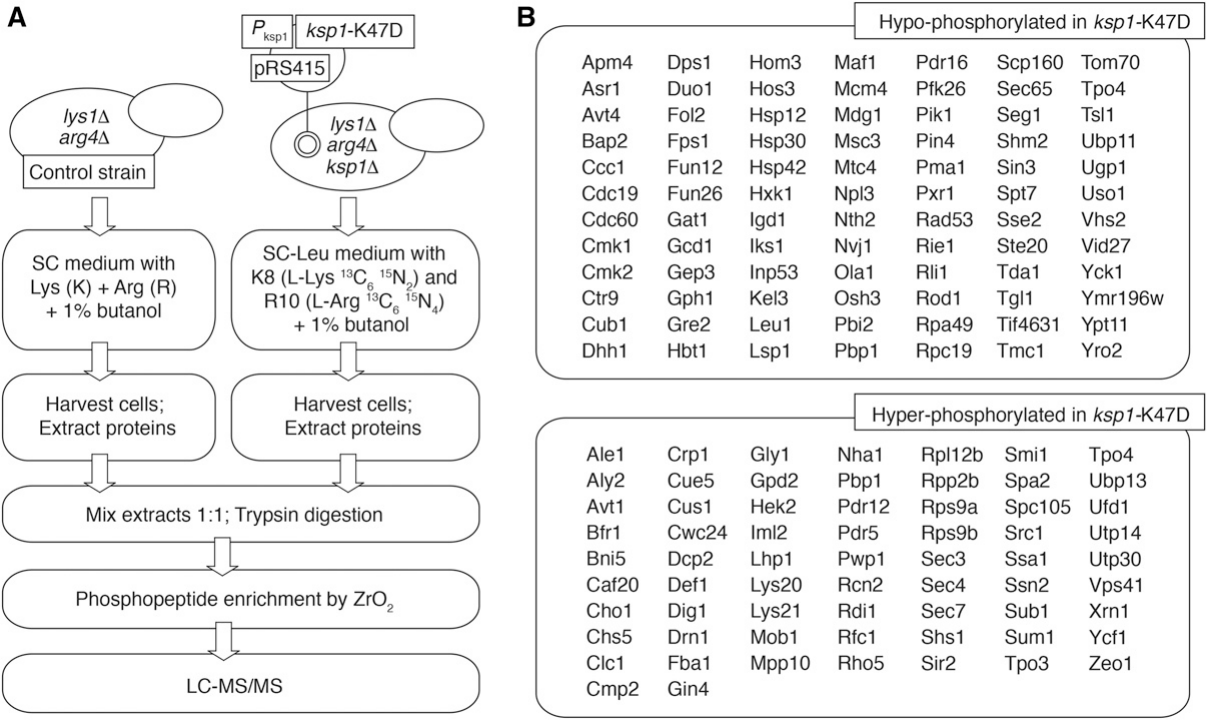
Quantitative phosphoproteomic analysis of Ksp1p kinase signaling. (A) Overview of the major steps for quantitative phosphoproteomics. The haploid yeast strain used for this analysis is deleted for the *LYS1* and *ARG1* genes. The kinase-defective *ksp1*-K47D allele with its native promoter was introduced on a low-copy centromeric plasmid (derived from pRS415) into a strain deleted for *KSP1*. Light and heavy variants of Lys and Arg are indicated. (B) Phosphopeptides corresponding to the listed proteins were identified as being hypo- or hyper-phosphorylated in the *ksp1*-K47D background. Standard names for each protein are provided when available.

For these studies, we utilized stable isotopic labeling with amino acids in cell culture (SILAC) and mass spectrometry to identify proteins differentially phosphorylated in a strain carrying *ksp1*-K47D (Ong *et al.* 2002; Ong and Mann 2006). For SILAC analysis, we generated a haploid strain of the filamentous Σ1278b background deleted for *LYS1* and *ARG4*, making the strain dependent upon exogenously supplied lysine and arginine for protein synthesis. We further deleted *KSP1* in this strain and introduced a low-copy, centromeric plasmid carrying the *ksp1*-K47D allele under transcriptional control of its native promoter. As indicated in Figure 3A, the control strain with wild-type *KSP1* was grown in media supplemented with light lysine and arginine, while the strain carrying *ksp1*-K47D was incubated in media with stable heavy isotopic forms of both amino acids. Filamentous growth was induced in both strains by the addition of the short-chain alcohol butanol. We selected this induction method because the presence of 1% butanol results in a very strong pseudohyphal response for haploid strains in liquid culture, forming extended filaments of elongated cells even without a solid substratum for adherence (Lorenz *et al.* 2000; Jin *et al.* 2008). After sufficient growth to ensure protein labeling, cells were harvested and phosphoproteins analyzed by liquid chromatography–tandem mass spectrometry. The approach is outlined in greater detail in Figure 3A.

By this approach, peptides corresponding to 84 proteins were identified as being hypo-phosphorylated relative to control in the strain with the *ksp1*-K47D allele, and 65 hyper-phosphorylated proteins were identified. It should be noted that by this approach, direct and indirect targets of Ksp1p kinase activity were identified collectively in the set of proteins hypo-phosphorylated in the *ksp1*-K47D mutant; presumably, indirect targets of Ksp1p kinase signaling are indicated by the hyper-phosphorylated proteins detected in *ksp1*-K47D. The full set of differentially phosphorylated proteins identified in this work is indicated in Figure 3B, and sites of differential phosphorylation in each protein are reported in the File S7.

**Figure 4:**
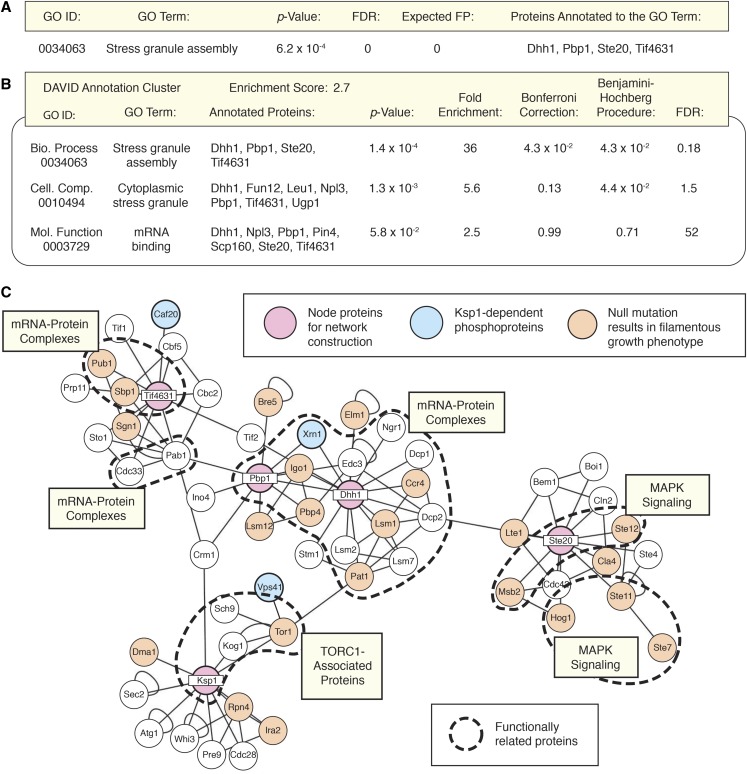
The Ksp1p-dependent phosphoproteome is enriched for proteins in ribonucleoprotein granules. (A) The GO Biological Process term “stress granule assembly” (GO: 0034063) is enriched in the set of proteins differentially phosphorylated in *ksp1*-K47D relative to the proteome as a whole. The annotated proteins are Dhh1p, Pbp1p, Ste20p, and Tif4631p. (B) Analysis with the DAVID software suite for data mining validates the above result, identifying enrichment of an annotation cluster with GO 0034063, the Cellular Component term “cytoplasmic stress granule” (GO: 0010494), and the Molecular Function term “mRNA binding” (GO: 0003729). (C) To identify the effect of signaling through these Ksp1p-dependent stress granule phosphoproteins, we constructed an interaction network using Dhh1p, Ksp1p, Pbp1p, Ste20p, and eIF4G/Tif4631p as nodes. Physical interactions extracted from BioGRID are indicated as lines. Ksp1p-dependent phosphoproteins identified in this study are shaded in blue, and proteins required for wild-type pseudohyphal growth are shown in orange. Subsets of functionally related proteins are outlined by dashed lines.

We analyzed the identified set of Ksp1p-dependent phosphoproteins for enrichment of functional annotations, including GO annotations. As shown previously in Figure 2, Ksp1p signaling is required for wild-type levels of gene transcripts functioning in amino acid biosynthesis and metabolism, relevant in establishing amino acid pools for translation. Consistent with this observation, the Ksp1p-dependent phosphoproteome is also enriched for proteins that regulate translation, but principally as components of cytoplasmic messenger ribonucleoprotein (mRNP) granules that are induced under conditions of cell stress (Figure 4). RNP stress granules are a type of membraneless organelle that forms via phase separation of translationally stalled/inactive RNA and proteins (Buchan and Parker 2009; Buchan *et al.* 2013). A statistically overrepresented set of mRNP stress granule proteins were identified as being hypo-phosphorylated in *ksp1*-K47D relative to a control strain. This set of mRNP granule-associated proteins in the Ksp1p-dependent phosphoproteome includes the helicase Dhh1p, the polyA-binding protein Pbp1p, the p21-activated signaling kinase Ste20p, and the eIF4G translation initiation factor Tif4631p (Buchan *et al.* 2010, 2011). Using the functional annotation tool DAVID to mine the set of proteins hypo-phosphorylated in *ksp1*-K47D, we identified additional Ksp1p-dependent phosphoproteins associated with cytoplasmic mRNP granules (Figure 4B). Using the identified core mRNP stress granule proteins to construct a protein-protein interaction network (Figure 4C), the resulting map suggests that Ksp1p signaling affects stress-responsive mRNP granules and that many granule proteins are required for wild-type pseudohyphal growth.

### Sites of Ksp1p-dependent phosphorylation in the stress granule proteins eIF4G/Tif4631p and Pbp1p are required for wild-type pseudohyphal growth

**Figure 5:**
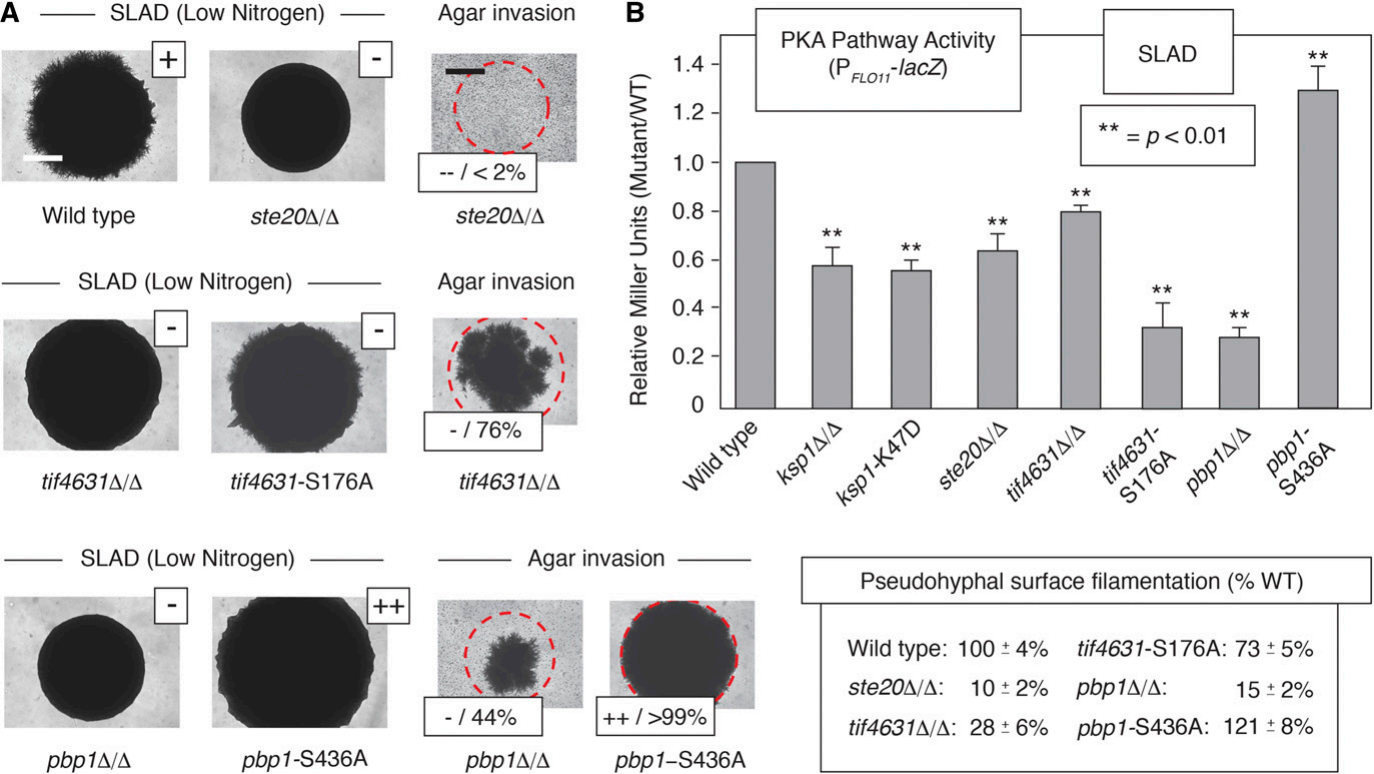
Ksp1p-dependent phosphorylation sites in the stress granule proteins eIF4G/Tif4631p and Pbp1p are required for wild-type pseudohyphal growth and PKA signaling through *FLO11*. (A) Surface filamentation phenotypes and agar invasion phenotypes are presented for wild type, *ste20*Δ/Δ, *tif4631* mutants, and *pbp1* mutants. Surface filamentation was assayed on medium with low levels of ammonium sulfate (SLAD). + indicates wild-type filamentation; − indicates decreased surface filamentation relative to wild type; ++ indicates increased surface filamentation relative to wild type. The *pbp1*-S436A mutant grew well beyond the boundaries of the original spotted culture and extended substantial surface filaments that formed a dense surrounding ring, the degree of which is beyond that observed in the wild-type strain. The degree of surface filamentation relative to wild type was quantified as described previously using the circumference (ImageJ) of a defined region from each spotted culture; percentages indicate the mean and SD from four replicate spots. Bar, 1 mm. Agar invasion was quantified for homozygous diploid mutants on low-nitrogen SLAD medium as the average pixel intensity for each spotted culture postwashing relative to the prewashed image as described previously. Wild-type agar invasion is presented in Figure 1. −−indicates severely decreased invasion relative to wild type; – indicates severely decreased invasion relative to wild type; ++ indicates increased agar invasion relative to wild type. Bar, 1 mm. (B) PKA pathway activity was estimated for the indicated homozygous diploid mutants in low-nitrogen SLAD media using a *lacZ* reporter driven by a region of the *FLO11* promoter regulated by PKA signaling. β-galactosidase levels are reported as the observed ratio relative to wild type.

To determine the pseudohyphal growth-related significance of stress granule proteins dependent upon Ksp1p for wild-type phosphorylation, we generated homozygous diploid strains in the filamentous Σ1278b background deleted for *DHH1*, *PBP1*, *STE20*, and *TIF4631*. Deletion of *DHH1* did not result in a pseudohyphal growth phenotype; however, loss of the other genes did affect filamentation (Figure 5).

*STE20* is required for wild-type pseudohyphal growth and agar invasion (Figure 5A). Ste20p belongs to the p21-activated kinase family and acts immediately upstream of the yeast filamentous growth Kss1p MAPK cascade, phosphorylating the MAPKKK Ste11p (Drogen *et al.* 2000). Ste20p also phosphorylates the stress granule-localized mRNA decapping protein Dcp2p, and Ste20p has been colocalized with stress granule proteins, including Pub1p-mCherry (Yoon *et al.* 2010; Mitchell *et al.* 2013). Notably, we observe that PKA pathway regulation of *FLO11* expression is decreased in homozygous diploid *ste20*Δ/Δ relative to wild type in low-nitrogen media, using a plasmid-based *lacZ* reporter fusion to a segment of the *FLO11* promoter regulated by the PKA pathway (Figure 5B). The reporter contains sequence corresponding to a region 1.0–1.4 kb upstream of the *FLO11* initiator ATG, encompassing binding sites for the PKA-regulated transcription factors Flo8p and Sfl1p (Aun *et al.* 2013). β-galactosidase levels from the FLO11*-lacZ* reporter are significantly depressed in a strain deleted for *FLO8* (Figure S3).

Tif4631p is an ortholog of the eukaryotic translation initiation factor eIF4G isoform 1, acting as a scaffold and interacting with poly(A)-binding protein and components of the mRNA cap-binding complex (Tarun *et al.* 1997; Gallie and Browning 2001). Tif4631p colocalizes with the stress granule marker Pub1p-mCherry, and a TAP-tagged form of the protein has been used to purify stress granule cores in yeast (Mitchell *et al.* 2013; Jain *et al.* 2016). We find that deletion of *TIF4631* in a homozygous diploid strain of filamentous *S. cerevisiae* results in decreased surface filamentation and invasive growth on medium with limited nitrogen (Figure 5A). Homozygous diploid *tif4631*Δ/Δ exhibits decreased PKA pathway activity relative to wild type under conditions of nitrogen limitation. Tif4631p undergoes Ksp1p-dependent phosphorylation at Ser176, as phosphorylation at this site is decreased in *ksp1*-K47D. Mutation of Tif4631p Ser176 to nonphosphorylatable alanine (S176A) results in decreased surface filamentation on low-nitrogen medium and decreased PKA pathway activity by the *FLO11* promoter-based reporter described above (Figure 5B). Steady-state levels of tif4631p-S176A tagged with the HA epitope at its carboxy terminus did not exhibit a significant change from observed levels of wild-type Tif4631p-HA under identical growth conditions (Figure S4A).

Pbp1p is an ortholog of poly(A)-binding protein, interacting with Pab1p to regulate mRNA polyadenylation (Mangus *et al.* 1998). Pbp1p is a component of the stress granule core substructure, and Pbp1p carboxy-terminal fluorescent protein fusions are routinely used as stress granule markers in yeast (Shah *et al.* 2013; Jain *et al.* 2016). Homozygous deletion of *PBP1* results in decreased surface filamentation and agar invasion relative to wild type on medium with low levels of ammonium sulfate as a nitrogen source (Figure 5A). Ser436 in Pbp1p is hyper-phosphorylated in a strain with the kinase-defective *ksp1*-K47D allele, suggesting an indirect mechanism of Ksp1p regulation at this residue. Mutation of Pbp1p Ser436 to alanine results in increased growth and agar invasion relative to wild type on low-nitrogen medium (Figure 5A). Consistent with this phenotype, the *FLO11* promoter-based *lacZ* reporter is hyperactive relative to wild type under conditions of nitrogen limitation in the homozygous *pbp1*-S436A mutant (Figure 5B). By Western blotting, levels of pbp1p-S436A tagged at its carboxy terminus with the HA epitope are not significantly different from levels of Pbp1p-HA (Figure S4B), indicating that the observed *pbp1*-S436A phenotypes are unlikely to result from altered expression of the point mutant.

### KSP1 regulates Pbp1p RNP granule abundance

**Figure 6:**
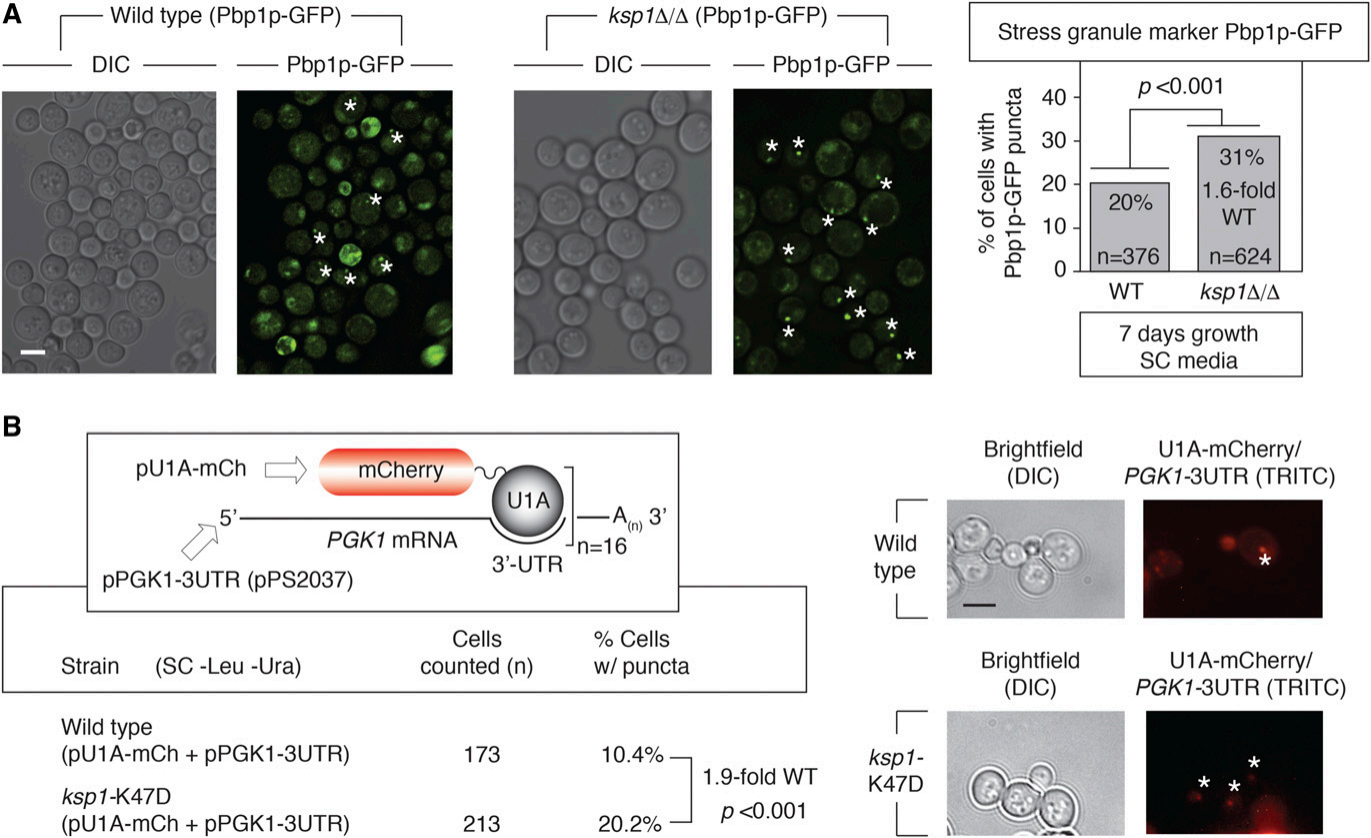
Loss of *KSP1* results in elevated ribonucleoprotein foci marked by Pbp1p. (A) Pbp1p was visualized as a carboxy-terminal fusion to GFP expressed from its native chromosomal locus in diploid wild-type or *ksp1*Δ/Δ strains. Asterisks indicate Pbp1p foci. The percentage of cells exhibiting Pbp1p foci was calculated for both strains. Cells were grown for an extended period of time in minimal media to induce high optical density/cell number stress. Consequently, cell autofluorescence, indicative of dead cells, can be seen in the image. The number of cells counted is indicated, and the percentages are indicated as a ratio relative to wild type, with 20% of cells in the wild-type strain exhibiting Pbp1p-GFP puncta. Bar, 2 μm. (B) The U1A-mCherry binding system was used to visualize mRNA in the homozygous diploid *ksp1*-K47D mutant. Cells were grown in SC media lacking leucine and uracil to maintain selection for the plasmids encoding U1A-mCherry and the modified *PGK1* gene. Cells were grown to high optical density as described above. Asterisks indicate U1A-bound mRNA puncta. The fold-increase of cells with puncta in the *ksp1*-K47D mutant relative to wild type is indicated along with the number of cells counted. Bar, 2 μm. *P*-values (<0.01)

RNP stress granule components are enriched above background in the set of proteins constituting the Ksp1p-dependent phosphoproteome, suggesting that Ksp1p may contribute to the regulation of stress-responsive RNP granules. Since the stress granule marker Pbp1p undergoes Ksp1p-dependent phosphorylation, we assessed its localization as a carboxy-terminal fusion to GFP in a homozygous diploid strain deleted of *KSP1*. As indicated in Figure 6A, Pbp1p-GFP foci are increased 1.6-fold relative to wild type in *ksp1*Δ/Δ. This phenotype is most evident upon growth to a high cell density in minimal medium, as Pbp1p foci are normally present but not evident in large numbers under this stress condition. We also observe a 1.5-fold increase in Pbp1p foci after 24 hr growth in media lacking glucose (Figure S2A). To corroborate this result, we determined the subcellular localization of mRNA transcripts corresponding to the housekeeping glycolytic enzyme Pgk1p using the system established by Brodsky and Silver (2000). For this approach, a plasmid encoding the human U1A RNA-binding protein as a fusion to mCherry and a plasmid encoding *PGK1* transcripts with U1A-binding sites incorporated into the 3′-UTR were introduced into *ksp1*Δ/Δ. Fluorescence microscopy of this strain indicated 3.3-fold increased mRNA puncta corresponding to the U1A-bound *PGK1* transcripts (Figure S2B). Using this U1A-based detection system, 1.9-fold increased mRNP foci were observed in the homozygous diploid *ksp1*-K47D mutant (Figure 6B).

**Figure 7:**
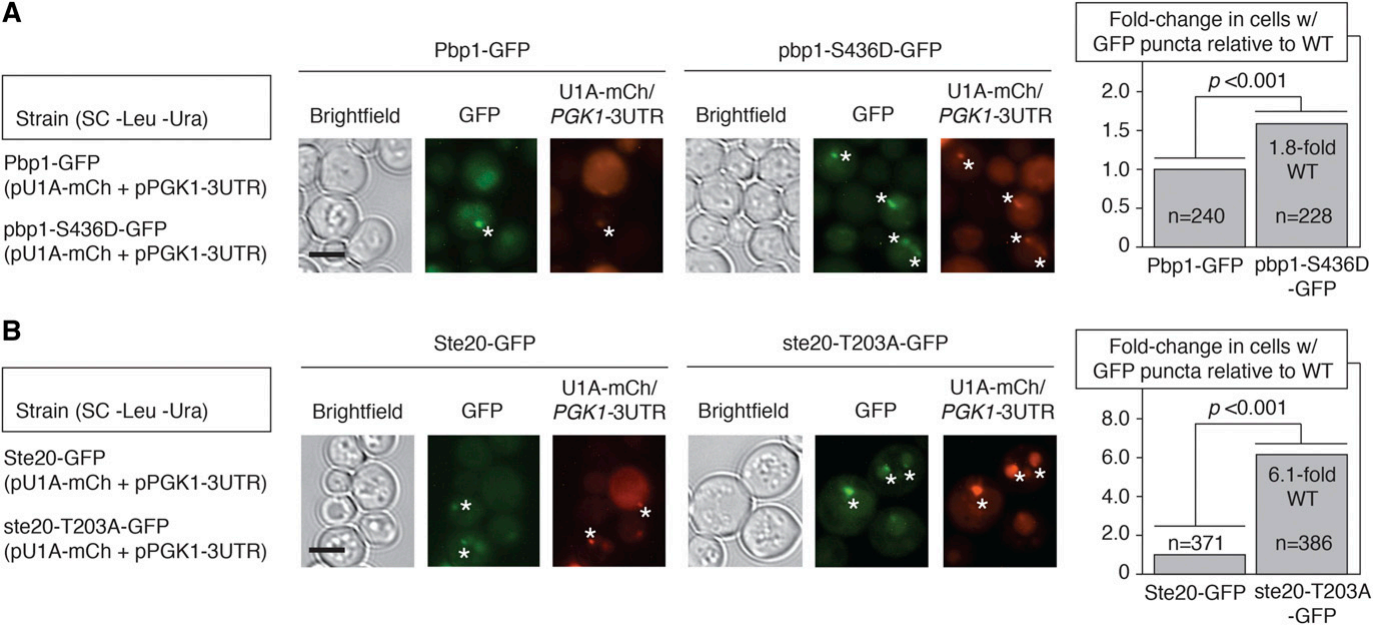
The Ksp1p-dependent Ser436 phosphorylation site in Pbp1p and Thr203 phosphosite in Ste20p are required for the wild-type localization of these respective stress granule-associated proteins. (A) A cassette encoding GFP was integrated at the 3′-end of wild-type *PBP1* and in the *pbp1*-S436D phosphomimetic mutant strains. Plasmids for visualization of modified *PGK1* mRNA by U1A-mCherry binding were introduced into each strain. Cells were grown to a high density by culturing the cells for an extended period of time in minimal media as described previously. Asterisks indicate Pbp1p-GFP puncta that also overlap with U1A-mCherry-bound mRNA. The fold-change in cells with GFP puncta relative to wild type is indicated along with the number of cells counted for each strain. Bar, 2 μm. (B) Chromosomal GFP-fusions were generated in a wild-type strain and in a strain with the *ste20*-T203A mutation by integration of the GFP-encoding cassette at the native *STE20* locus as described above. Cells were cultured, imaged, and quantified for GFP and U1A-bound mRNA puncta as indicated above. Wild-type Ste20p puncta are not as easily visualized as Pbp1p puncta (observed in 1% of cells); consequently, the fold-change increase in puncta observed for the *ste20*-T203A mutant is more dramatic. Bar, 2 μm. *P*-values (<0.01)

Since Tif4631p, Pbp1p, and Ste20p are stress granule-localized proteins that undergo Ksp1p-dependent phosphorylation, we determined the subcellular localization of each protein with its respective Ksp1p-dependent phosphorylation site mutated. A GFP fusion to the carboxy terminus of Tif4631p-S176A exhibited a wild-type punctate localization pattern under normal growth conditions and under conditions inducing stress granule assembly. Pbp1p is hyper-phosphorylated at Ser436 in *ksp1*-K47D; consequently, we generated a phosphomimetic Ser-to-Asp substitution to determine the effect of phosphorylation at this site on its subcellular localization. As indicated in Figure 7A, the Pbp1p-S436D-GFP chimera expressed from its native chromosomal locus in the *ksp1*-K47D background exhibited 1.8-fold elevated foci abundance relative to a wild-type allele of *PBP1*. The Ste20p kinase is phosphorylated in a Ksp1p-dependent fashion at Thr203, with the residue hypo-phosphorylated in *ksp1*-K47D. A carboxy-terminal GFP fusion to a mutated form of this protein with Ala swapped for Thr203 indicates 6.1-fold elevated Ste20p-containing RNP granules (Figure 7B). Consistent with data observed in the fluorescent micrographs, Western blotting indicates that total protein levels of HA-tagged ste20p-T203A-HA are not significantly different from levels of Ste20p-HA (Figure S4A). Collectively, the data are consistent with an observed increase in stress granule RNP foci in a yeast strain defective for Ksp1p signaling. The data further indicate that Ksp1p-dependent phosphorylation regulates the localization of Pbp1p and Ste20p in RNP foci.

## Discussion

The Ksp1p kinase is a putative effector of PKA and TORC1 signaling, but the purview of its regulatory control in eukaryotic stress responses has been largely unclear. Here, we used transcriptional profiling and quantitative phosphoproteomics to determine cellular processes regulated through Ksp1p signaling. Transcript levels for genes functioning in pathways for amino acid biosynthesis and metabolism were perturbed in a filamentous yeast strain carrying the kinase-defective *ksp1*-K47D allele. Phosphorylation of a statistically significant set of translation-associated proteins in RNP granules was altered in this mutant. Amino acid metabolism and RNP function contribute to the translational control and repression evident in response to nitrogen limitation. The Ksp1p-dependent phosphorylation sites Ser176 in Tif4631p and Ser436 in the stress granule marker Pbp1p are required for wild-type pseudohyphal growth. Loss of *KSP1* results in elevated Pbp1p-marked RNP granules, consistent with a function for Ksp1p in regulating stress granule abundance.

The studies here indicate that Ksp1p kinase signaling contributes to the regulation of many eukaryotic stress responses. Ksp1p is required for pseudohyphal growth, and the Ksp1p-dependent signaling network is enriched in genes contributing to relevant processes, including cell wall organization, DNA replication, and the cell morphogenesis checkpoint. GO terms related to these processes were enriched in previous gene disruption and overexpression screens for pseudohyphal growth regulators (Jin *et al.* 2008; Ryan *et al.* 2012; Shively *et al.* 2013). Genes required for cell-cell adhesion are also enriched in the set of transcripts regulated through Ksp1p signaling. Extending beyond processes intimately associated with pseudohyphal growth, genes mediating the cellular response to oxidative stress, autophagy genes, and genes required for sporulation undergo changes in transcript abundance in a strain of yeast with defective Ksp1p kinase activity. This broad function for the Ksp1p regulatory network is consistent with its role in PKA and TORC1 signaling. TORC1 has been proposed to control PKA activity toward selective substrates, and the molecular basis of the Ksp1p signaling network suggests that it may contribute to the integration of PKA and TORC1 signaling, possibly toward the PKA regulatory subunit Bcy1p, which is mislocalized in a *ksp1* loss-of-function mutant (Bharucha *et al.* 2008). Control of *FLO11* levels by the PKA-regulated Flo8p and Sfl1p transcription factors is altered in *ksp1* mutants and in strains with mutated Ksp1p-dependent phosphosites in *TIF4631* and *PBP1*, although it should be noted that signaling pathways beyond PKA may contribute to the regulation of these transcription factors.

Analysis of the Ksp1p-dependent phosphoproteome was notable in that it identified a significant set of proteins localized to stress granules, including proteins in the translation initiation closed-loop complex, such as eIF4G/Tif4631p, or proteins that bind to this complex, such as Pbp1p and Caf20p. Tif4631p has been immunoprecipitated as part of the proteinaceous stress granule core (Jain *et al.* 2016). Its Ksp1p-dependent phosphorylation site (Ser176) has not been reported previously and does not lie in a region of Tif4631p corresponding to a known protein domain. Tif4631p Ser176 is required for wild-type pseudohyphal growth but is dispensable for the regulation of stress granule abundance. Tif4631p has been identified previously as an *in vitro* substrate of Ksp1p (Chang and Huh 2018). The substrate specificity of Ksp1p is unknown, and a substrate recognition motif is not evident in the protein set identified from the phosphoproteomic studies reported here. Consequently, it is unclear if Ser176 is directly phosphorylated by Ksp1p. The observation that Tif4631p Ser176 is required for wild-type pseudohyphal growth but dispensable for its subcellular localization suggests that separate Ksp1p outputs may regulate pseudohyphal growth and RNP abundance. Pbp1p is also a core stress granule protein, and its regulation by Ksp1p is more complex, as Ser 456 is hypo-phosphorylated in *ksp1*-K47D, while Ser436 is hyper-phosphorylated in this mutant background. Both phosphorylation sites have been identified previously (Holt *et al.* 2009; Soulard *et al.* 2010), although we did not observe any pseudohyphal growth phenotypes for Ser456. The Ser436 site is indirectly regulated through Ksp1p signaling. Notably, the phosphomimetic Pbp1p-S436D mutant forms increased RNP granules, similar to the phenotype observed in *ksp1*-K47D. The Ser436 site in Pbp1p does not conform to the consensus substrate motif for a known signaling kinase, and additional work will be needed to identify its direct regulatory kinase. The data here indicate that Ksp1p kinase signaling results in the phosphorylation of Tif4631p at Ser176 and the dephosphorylation of Pbp1p at Ser436. Both phosphorylation events contribute to the inhibition of RNP granule proliferation.

Recent studies suggest that the regulation of stress granule proliferation and disassembly may be achieved through protein-protein and protein-RNA interactions, with proteins containing prion-like, intrinsically disordered regions contributing significantly to these interactions (Wheeler *et al.* 2016). Ksp1p was identified in biochemically purified yeast stress granules, and its sequence is predicted to encode a disordered RNA-binding region (Jain *et al.* 2016). Ksp1p was also identified experimentally as an RNA-binding protein by UV cross-linking and mass spectrometric analysis of proteins from purified RNA-protein complexes (Mitchell *et al.* 2013). Stress granule core proteins have been hypothesized to nucleate granule assembly; however, stress granule abundance is increased upon loss of *KSP1*. Consequently, Ksp1p may be a noncanonical stress granule core protein that either inhibits excessive stress granule assembly or facilitates granule disassembly. Studies of RNP dynamics will be necessary to distinguish between these possibilities.

There is precedence for signaling kinases integrating the pseudohyphal response and RNP granule dynamics. We previously colocalized the yeast MAPKs Kss1p and Fus3p and the PKA catalytic subunit Tpk2p with the stress granule and P-body–localized protein Igo1p. Deletion of *KSS1* disrupted RNP foci under conditions of glucose limitation, as visualized using the U1A RNA-binding platform (Shively *et al.* 2015). In yeast, PKA inhibits the formation of large P-body aggregates by phosphorylating the granule scaffolding protein Pat1p (Ramachandran *et al.* 2011). The degree to which Ksp1p and the signaling kinases above regulate translation through the control of stress granule abundance is an open question. Polyribosome profiling of mutants defective in stress granule assembly does not show obvious phenotypes, which leaves the likely possibility that the translation of specific key transcripts may be regulated through RNP trafficking. Similar analyses may need to be performed for cells exposed to various stresses to assess the mechanistic basis of stress granule and RNP function. The work here underscores the importance of stress-responsive signaling pathways in regulating the abundance of RNPs, while providing a molecular basis for Ksp1p signaling as a node in the eukaryotic stress response network.
